# Zinc transporter 8 (ZnT8) autoantibody prevalence in black South African participants with type 1 diabetes

**DOI:** 10.1186/s12902-021-00812-8

**Published:** 2021-07-16

**Authors:** Sureka Bhola, Eleanor M Cave, Sindeep Bhana, Nigel J Crowther, Carolyn J Padoa

**Affiliations:** 1 Department of Chemical Pathology, Faculty of Health Sciences, University of the Witwatersrand, Johannesburg, South Africa; 2 National Health Laboratory Service, Johannesburg, South Africa; 3 Department of Medicine, Chris Hani Baragwanath Hospital, Faculty of Health Sciences, University of the Witwatersrand, Johannesburg, South Africa

**Keywords:** ZnT8, Autoantibody, Type 1 diabetes, Black South African, Prevalence

## Abstract

**Background:**

Autoantibodies to β-cell specific antigens are markers of type 1 diabetes. The most recently identified autoantibodies are targeted to the zinc transporter 8 (ZnT8) protein located in the membrane of β-cell insulin secretory granules. The prevalence of ZnT8 autoantibodies in newly diagnosed participants with type 1 diabetes has been found to range from 33 to 80 %. Due to the lack of data on the immunological aetiology of type 1 diabetes in African populations, this study aimed to determine the prevalence of ZnT8 autoantibodies in black South Africans with type 1 diabetes and whether ZnT8 autoantibody positivity was associated with age at diagnosis and disease duration.

**Methods:**

Participants with type 1 diabetes and controls were recruited from the greater Johannesburg area, South Africa. Positivity for ZnT8, GAD65 and IA2 autoantibodies was determined by ELISA.

**Results:**

Participants with type 1 diabetes (n = 183) and controls (n = 49) were matched for age (29.1 ± 9.53 vs. 27.3 ± 7.29, respectively; p = 0.248). The mean age at diagnosis for participants with type 1 diabetes was 20.8 ± 8.46 years. The prevalence of ZnT8 autoantibody positivity was 17.5 % (32 of 183) in participants with type 1 diabetes with a median disease duration of 7.00 [2.00; 11.0] years. ZnT8 autoantibody prevalence in newly diagnosed participants (< 1 year duration) was 27.3 % (6 of 22). Logistic regression analysis found an association between ZnT8 autoantibody positivity and shorter disease duration (OR: 0.9 (0.81-1.00); p = 0.042). In addition, ZnT8 autoantibody positivity was significantly associated with an increased chance of being GAD65 (OR: 3.37 (1.10–10.3)) and IA2 (OR: 8.63 (2.82–26.4)) autoantibody positive. Multiple regression analysis found no association between ZnT8 autoantibody positivity and age at diagnosis. However, the presence of ≥ 2 autoantibodies was associated with a younger age at diagnosis of type 1 diabetes when compared to participants with ≤ 1 autoantibody (B = -5.270; p = 0.002).

**Conclusions:**

The presence of ZnT8 autoantibodies was not related to a younger age at diagnosis in black South African patients with type 1 diabetes. However, the greater the numbers of autoantibodies present in an individual the earlier the age at diagnosis. ZnT8 autoantibodies decline with disease duration in the black South African population.

## Background

Type 1 diabetes (T1D) is a chronic autoimmune disease which results in the destruction of the insulin secreting β-cells in the pancreatic islets of Langerhans [1]. The disease is characterised by the presence of autoantibodies (AAbs) to beta-cell specific antigens which may develop years before the clinical diagnosis of the disease [2, 3]. Autoantibodies directed to the zinc transporter 8 (ZnT8) peptide have been described in T1D [4]. This peptide is encoded by the *SLC308* gene located on chromosome 8q24.11 and is found in the membrane of insulin secretory granules located in pancreatic β-cells [5]. The ZnT8 peptide mediates the uptake of Zn^2+^ into the secretory granules which in turn stabilises insulin by allowing hexamer formation [6, 7].

Several studies have determined the prevalence of ZnT8 AAb positivity in T1D, which varies with ethnicity. In Europeans, the prevalence of ZnT8 AAbs ranged between 60 and 80 % of newly diagnosed participants with T1D [4, 8–11]. In comparison, Asian populations reported relatively low ZnT8 AAb prevalences in newly diagnosed individuals with T1D, with a Chinese and a Japanese study reporting prevalences of 32.9 % (48 of 146) and 36 % (40 of 112), respectively [12, 13]. The T1D Genetics Consortium found that in newly diagnosed non-Hispanic black participants (duration < 3 years) the ZnT8 prevalence was 57.9 % (Wenzlau et al., 2015).

The prevalence of ZnT8 AAbs has been shown to decline with increasing disease duration. A study by Howson et al., [14] conducted in British participants with T1D showed that ZnT8 AAb positivity decreased from 58 % in participants with a disease duration ≤ 2 years to 10 % in those with a disease duration ≥ 9 years. Fabris and colleagues found that in an Italian study, the prevalence of ZnT8 AAbs remained stable for the first 4 years after diagnosis of T1D (61.1 % at onset, 56.0 % at 1 year and 59.3 % at 2–4 years) and then dropped significantly to 33.8 % at ≥ 5 years [10]. Similar declines in ZnT8 AAb frequency with increasing disease duration were seen in studies conducted in Brazilian, Japanese and non-Hispanic white populations [9, 15, 16].

The ZnT8 AAbs are more prevalent in newly diagnosed children than adults with T1D. Thus, a Swedish study found the prevalence of ZnT8 AAb positivity to be 66 % (227 of 343) in newly diagnosed participants with an age at onset between 15 and 34 years compared to 80 % (200 of 249) in a younger cohort with an age at onset of 2–17 years [8]. In a Polish cohort of newly diagnosed individuals with T1D, ZnT8 AAbs were more prevalent in children (81.1 % [177 of 218], median age 9 years [interquartile range: 6–13 years]) than in adults (34.8 % [52 of 149], median age 34 years [interquartile range: 27–43 years]) [17]. Similar associations of ZnT8 AAb positivity with younger age at diagnosis have been observed in Asian populations [12, 16].

Few studies have assessed the frequency of ZnT8 AAb positivity in sub-Saharan African populations. In a cohort of 85 newly diagnosed Ugandan individuals with T1D (< 25 years old), the prevalence of ZnT8 AAb positivity was 17.6 % [18]. A study conducted on newly diagnosed Ethiopians with T1D (≤ 35 years) found an overall frequency of 10.2 % (24 of 236) for ZnT8 AAbs. Although there was a decline in ZnT8 AAb positivity with an increasing age at diagnosis it did not reach significance (0–15 years: 16.7 % (6 of 36) vs. 16–35 years: 9.0 % (18 of 200, *p* = 0.161) [19]. A study conducted in Somalian migrants living in America found a ZnT8 AAb prevalence of 26 % (8 of 31) in participants with T1D with an age at onset ≤ 19 years [20].

Due to the lack of studies on ZnT8 AAbs in T1D in sub-Saharan Africa, this study aimed to determine the prevalence of ZnT8 AAbs in black South Africans with T1D. In addition, we aimed to determine whether ZnT8 AAb positivity was associated with age at diagnosis and disease duration.

## Methods

### Participant recruitment

Clinically diagnosed black South African participants with T1D (cases; n = 183) were recruited from diabetes clinics at Chris Hani Baragwanath Academic Hospital and Charlotte Maxeke Johannesburg Academic Hospital between November 2014 and December 2015. The study protocol was approved by the University of the Witwatersrand Human Research Ethics Committee (clearance certificate numbers M180334 and M150885) and by the South African Blood Transfusion Services Research Ethics Committee (clearance certificate number 2014/19). The study protocol conformed to the Declaration of Helsinki ethical guidelines. Informed written consent was obtained from all participants prior to commencement of the study. Black participants that were not diabetic (controls; n = 49) were recruited from the South African Blood Transfusion Services blood drives and from students and staff based at the University of Witwatersrand Medical School in Johannesburg. Participants were classified as controls if they had a random plasma blood glucose level < 11.1mmol/L and were not on any glucose lowering medication. Anthropometric measurements were taken for all participants. Patient glycated haemoglobin (HbA1c) and glucose levels were obtained from patient files. Participants with clinical evidence of chronic pancreatitis, gestational diabetes or type 2 diabetes were excluded from the study.

### Measurement of glucose concentrations

Random plasma glucose concentrations for control participants were measured on the ADVIA Chemistry System (Siemens Health Care Diagnostics Inc., New York, USA) in the National Health Laboratory Services Chemical Pathology Diagnostic Laboratory based at Charlotte Maxeke Johannesburg Academic Hospital using the enzymatic hexokinase method.

### Measurement of ZnT8, GAD65 and IA2 AAbs

The ZnT8, 65 kDa isoform of glutamic acid decarboxylase (GAD65) and protein tyrosine phosphatase related islet antigen 2 (IA2) AAb status was determined using ELISA kits (KRONUS, Idaho, USA) according to the manufacturer’s instructions. Based on the manufacturer’s guidelines, participants with ZnT8 and IA2 AAb concentrations ≥ 15 U/ml, and GAD65 AAb concentrations ≥ 5 IU/ml were classified as positive.

### Statistical analysis

Continuous data that were not normally distributed were log transformed to normality with the exception of duration of disease for which the square-root was used. Normally distributed continuous variables were presented as mean ± standard deviation (SD) whilst skewed data were presented as median [lower quartile; upper quartile]. Categorical variables were presented as percentages (%). The two-tailed Student’s non-paired t-test was used to compare variables between participants with T1D who were AAb positive and those who were AAb negative. A chi squared (χ^2^) test was used to compare all categorical variables. Backwards, stepwise multivariable logistic regression analyses were performed using ZnT8 AAb positivity as the dependent variable and independent variables chosen based on scientific plausibility and their association (*p* < 0.20) with AAb positivity in univariate analyses. Backward stepwise multivariable linear regression analysis was similarly performed with age at diagnosis as the dependent variable. Results with a *p* value < 0.05 were considered to be statistically significant. All statistical analyses were performed using Statistica software version 13 (StatSoft, Tulsa, Oklahoma, USA).

## Results

### Clinical and phenotypic characteristics of patients with T1D and control participants

The clinical and phenotypic characteristics of the study participants are summarised in Table 1. The patients with T1D had significantly more males (54.6 vs. 34.7 %; *p* = 0.013) and had higher glucose levels (9.20 [5.70; 13.2] vs. 5.00 [4.50; 6.00] mmol/L; *p* < 0.001) and a higher frequency of ZnT8 (17.5 vs. 2.0 %; *p* = 0.006) and GAD65 AAb positivity (51.3 vs. 2.1 %; *p* < 0.001) when compared to controls. The BMI was significantly higher in the control group than in the patient group (25.4 [23.4; 28.2] vs. 23.7 [21.2; 27.8];* p* = 0.023). The mean age at diagnosis of T1D was 20.8 ± 8.46 years with a median duration of disease of 7.00 [2.00; 11.0] years.

**Table 1:** Clinical and phenotypic characteristics of the study participants

Variables	Cases (n = 183)	Controls (n = 49)	*p* value
Age (years)	29.1± 9.53	27.3 ± 7.29	0.248
Age at diagnosis (year)	20.8 ± 8.46	-	-
Duration of disease (years)	7.00 [2.00; 11.0]	-	-
Gender			
males, % (n)	54.6 (100)	34.7 (17)	0.013
Females, % (n)	45.4 (83)	65.3 (32)	
Glucose (mmol/L)	9.20 [5.70; 13.2]^a^	5.00 [4.50; 6.00]	<0.001
HbA1c (%)	10.6 ± 3.41	-	-
BMI (kg/m^2^)	23.7 [21.2; 27.8]	25.4 [23.4; 28.2]	0.023
ZnT8 AAb positivity (%; n)	17.5 (32)	2.0 (1)	0.006
GAD65 AAb positivity (%; n)	51.3 (80)^b^	2.1 (1)^c^	<0.001
IA2 AAb positivity (%; n)	12.8 (20)^b^	6.5 (3)^d^	0.237

Results are presented as median values [lower quartile; upper quartile] for skewed data and as mean ± standard deviation for non-skewed data and % (n) for categorical variables; Missing data: ^a^n = 14, ^b^n = 27, ^c^n = 2, ^d^n = 3

### Autoantibody positivity, age at diagnosis and disease duration

The association of clinical characteristics in the participants with T1D with ZnT8 AAb positivity can be seen in Table 2. The ZnT8 AAb positivity was associated with a younger age at diagnosis (17.0 ± 6.49 vs. 21.6 ± 8.62 years, *p* = 0.005), a shorter duration of disease (3.50 [1.00; 8.00] vs. 7.00 [3.00; 12.0] years; *p* = 0.003), higher glucose concentrations (10.9 [7.10; 16.1] vs. 8.40 [5.40; 13.1] mmol/L; *p* = 0.042) and a lower BMI (20.1 [19.4; 23.9] vs. 23.9 [21.4; 28.7] kg/m^2^; *p* = 0.003). In addition, ZnT8 AAb positive participants had significantly higher IA2 (44.4 vs. 6.2 %; p <0.001) and GAD65 (81.5 vs. 45.0 %; *p* <0.001) AAb positivity when compared to ZnT8 AAb negative participants.

**Table 2:** Association of ZnT8 AAb positivity with clinical and phenotypic variables in participants with T1D

Variable	ZnT8 AAb positivity		*p* value
	Positive (n=32)	Negative (n=151)	
Age at diagnosis (years)	17.0 ± 6.49	21.6 ± 8.62	0.005
Duration of disease (years)	3.50 [1.00; 8.00]	7.00 [3.00; 12.0]	0.003
Gender			
Males, % (n)	59.4 (19)	53.6 (81)	0.696
Females, % (n)	40.6 (13)	46.4 (70)	
Glucose (mmol/L)	10.9 [7.10; 16.1]^a^	8.40 [5.40; 13.1]^b^	0.042
HbA1c (%)	10.9 ± 3.50	10.5 ± 3.40	0.593
BMI (kg/m^2^)	20.1 [19.4; 23.9]	23.9 [21.4; 28.7]	0.003
IA2 AAb positivity (%; n)	44.4 (12)^c^	6.2 (8)^d^	<0.001
GAD65 AAb positivity (%; n)	81.5 (22)^c^	45.0 (58)^d^	<0.001

Results are presented as median values [interquartile range] for skewed data and as mean ± standard deviation for non-skewed data; Missing data: ^a^n = 4, ^b^n = 10, ^c^n = 5 and ^d^n = 22

There was no significant difference in ZnT8 and IA2 AAb frequencies within the first three years following T1D diagnosis (*p* = 0.961, p = 0.729, respectively), whereas significant differences were observed for GAD65 AAb positivity (*p* = 0.049). However, there was a significant decline in ZnT8 and GAD65 AAb frequencies between participants with a disease duration ≤ 3 years and those with a disease duration ≥ 4 years (25.4 vs. 13.4 %; *p* = 0.044 and 66.7 vs. 44.2, *p* = 0.009, respectively) (Table 3). However, the median ZnT8 AAb titres in participants who tested positive for this AAb and with disease duration ≤3 years (173.4 [42.7; 227.2] U/ml) was not significantly different from those with disease duration ≥ 4 years (125.2 [76.2; 403.7] U/ml; *p* = 0.580).

**Table 3:** Autoantibody frequency in patients with T1D according to duration of disease

	Duration of disease						
	< 1 year (n=22)	1-2 years (n=28)	3 years (n=13)	p value	≤ 3 years (n=63)	≥ 4 years (n=119)	*p* value
ZnT8 AAb positivity (%, n)	27.3 (6)	25.0 (7)	23.1 (3)	0.961	25.4 (16)	13.4 (16)	0.044
GAD65 AAb positivity (%, n)	50.0 (9)^a^	85.7 (18)^b^	58.3 (7)^c^	0.049	66.7 (34)^d^	44.2 (46)^e^	0.009
IA2 AAb positivity (%, n)	16.7 (3)^a^	19.0 (4)^b^	0.00 (0)^c^	0.729	13.7 (7)^d^	12.5 (13)^e^	0.831

Missing data: ^a^n = 3, ^b^n = 7, ^c^n = 1, ^d^n = 12, ^e^n = 15

The greater the number of AAbs present in an individual, the younger the age at diagnosis (*p* = 0.008) and the shorter the disease duration (*p* = 0.022) (Table 4). In addition, the presence of AAbs (irrespective of number) was associated with a lower BMI (*p* = 0.040).

**Table 4:** Associations of number of autoantibodies and clinical characteristics in patients with T1D

Variable	0 AAbs (n = 69)	1AAb (n = 57)	2 or 3 AAbs (n = 30)	*p* value
Age at diagnosis (years)	22.6 ± 8.00^a^	21.4 ± 9.62^a^	16.9 ± 6.02	0.008
Duration of disease (years)	8.00 [4.00; 13.0]^a^	5.00 [2.00; 12.0]	4.00 [1.00; 8.00]	0.022
BMI (kg/m^2^)	25.2 [21.8; 29.9]^a^	22.5 [20.3; 25.3]^a^	22.9 [21.6; 25.7]^a^	0.040
Glucose (mmol/L)	8.25 [5.15; 13.0]^b^	8.10 [5.40; 13.1] ^b^	10.9 [5.80; 17.1]^c^	0.249
HbA1c (%)	9.87 ± 3.14^d^	10.8 ± 3.32^c^	10.6 ± 3.46^e^	0.255

Results are presented as median values [interquartile range] for skewed data and as mean ± standard deviation for non-skewed data; Missing data: ^a^n = 1, ^b^n = 5, ^c^n = 4, ^d^n = 6, ^e^n = 2

In order to identify the main determinants of ZnT8 AAb positivity, relevant variables that associated with the presence of these AAbs at *p* < 0.20 (i.e. disease duration, BMI, GAD65 and IA2 AAb positivity; see Table 2) were included in a backward stepwise multivariate logistic regression analysis (Table 5). This showed that ZnT8 AAb positivity was associated with a shorter disease duration (OR (95 % CIs): 0.90 (0.81-1.00); *p* = 0.042). In addition, both GAD65 and IA2 AAb positivity were associated with ZnT8 AAb positivity (*p* = 0.033 and *p* < 0.001, respectively).

**Table 5:** Logistic regression model for the determinants of ZnT8 AAb positivity

Dependent variable	Independent variable	Odds ratio (95% CI)	*p* value
ZnT8 AAb positivity	Disease duration	0.90 (0.81-1.00)	0.042
	GAD65 AAb^a^	3.37 (1.10-10.3)	0.033
	IA2 AAb^b^	8.63 (2.82-26.4)	<0.001

*For full model* *p* < 0.001 (n = 155), ^a^GAD65 positive = 1, GAD65 negative = 0; ^b^IA2 positive = 1, IA2 negative = 0

The possible determinants of age at diagnosis were identified using a backward stepwise multivariable linear regression analysis using relevant independent variables that correlated with age at diagnosis in a Pearson correlation at *p* < 0.20 (i.e. gender, ZnT8, GAD65 and IA2 AAb positivity; Table 6). In this model ZNT8 AAb positivity was not significantly associated with age at diagnosis, with only IA2 AAb positivity showing a significant negative association (*p* = 0.005) and gender tending toward significance (*p* = 0.052)

**Table 6:** Multiple regression model for the determinants of age at diagnosis

Dependent variable	Independent variable	B value	*p* value
Age at diagnosis	IA2 AAb positivity ^a^	-5.646	0.005
	Gender^b^	2.639	0.052

^*a*^*IA2 AAb code 0 = negative a 1 = positive;*^*b*^*Gender code 0 = female and 1 = male; for full model* *p* < 0.005 (n = 154); R^2^ = 0.056

To determine whether the number of AAbs present affected age at diagnosis, backward stepwise multivariable linear regression analysis controlling for gender was performed (Table 7). The presence of two or more AAbs was associated with an age at diagnosis five years younger than participants with zero or one AAb (*p* = 0.002). This model accounted for 6.7 % of the factors contributing to age at diagnosis.

**Table 7:** Multiple regression model for the determinants of age at diagnosis

Dependent variable	Independent variable	B value	*p* value
Age at diagnosis	>1 AAb^a^	-5.270	0.002
	Gender^b^	2.539	0.059

^*a*^*>1 AAb code: 0 = <2 AAbs present 1 = >2 AAbs;*^*b*^*Gender code: 0 = female and 1 = male for full model* *p* < 0.002 (n = 154); R^2^ = 0.067

## Discussion

There is a paucity of data regarding the prevalence of T1D-associated AAbs in sub-Saharan African populations. In the current study, the prevalence of ZnT8 AAbs in black South Africans with T1D (median disease duration of seven years) was 17.5 % whereas in the control population the prevalence was only 2 %. In newly diagnosed participants (< 1 year duration) the prevalence was 27.3 %). When comparing participants with a disease duration ≤ 3 years to those with a longer disease duration, ZnT8 and GAD65 AAb frequency declined (*p* = 0.044 and 0.009, respectively). The association of ZnT8 AAb positivity and disease duration was confirmed in a logistic regression model (*p* = 0.042). Participants who were GAD65 and IA2 positive had a 3.37 and an 8.63 times increased likelihood of being ZnT8 positive, respectively. In participants with T1D, ZnT8 AAb positivity was associated with a younger age at diagnosis, however, this association was lost in regression analysis whereas IA2 AAb positivity remained associated with a younger age at diagnosis (*p* = 0.005). In addition, a younger age at diagnosis was found in participants with ≥ 2 AAbs compared to those with ≤ 1 AAbs. To our knowledge, this is the first report of ZnT8 AAbs in black South Africans with T1D.

The overall prevalence of ZnT8 AAb positivity (17.5 %; median duration 7 years) in our cohort was lower but not significantly (*p* > 0.05) different to that in a Chinese population (24.1 % (130 of 539), median disease duration of 2 (range 0–348) months and Somalian immigrants living in America (25.8 % (8 of 31), median disease duration of 3.1 years) [12, 20]. However, our population had significantly (*p* < 0.05) lower frequencies of ZnT8 AAb than Japanese (28 % (75 of 270), median disease duration of 2 years), and British (33.6 % (752 of 2237), median disease duration of 4 years) populations [13, 14]. In our cohort, the prevalence of ZnT8 AAbs in newly diagnosed (< 1 year) black South Africans with T1D (27.3 %, 6 of 22) is higher but not significantly (*p* > 0.05) different to that reported in a Ugandan cohort newly diagnosed with T1D (17.6 % (15 of 85)) [18], but is significantly (*p* < 0.05) higher than ZnT8 AAb frequencies reported in newly diagnosed Ethiopians with T1D (10.2 % (24 of 236), *p* = 0.017), and significantly lower than non-Hispanic blacks (62.2 % (46 of 74); *p* = 0.004) and non-Hispanic whites (67.2 % (180 of 268); *p* < 0.001) [9, 19]. A longitudinal study (4–12 year follow up) conducted in a predominantly white cohort showed that 50 % of participants with T1D seroconverted from ZnT8 AAb positive to negative status after a disease duration of 6.2 years [21]. Thus, as the median duration seen in our study is 7 years, it is likely that the 17.5 % ZnT8 AAb prevalence is an underestimation, and this is confirmed by the higher prevalence of ZnT8 AAbs (27.3 %) observed in participants with a disease duration of < 1 year. These studies suggest that the frequency of the ZnT8 AAb is lower in indigenous African populations.

Previous studies involving both cross-sectional and longitudinal analyses [8, 21, 22] have shown that ZnT8 AAb titres decline with increasing duration of disease. In the current study, we did not find a difference in ZnT8 AAb titres when comparing individuals with varying disease duration, but this may be a reflection of our small sample size and/or due to the type of assay used i.e. ELISA or radiobinding assays, as their sensitivity and specificity are known to differ [23].

The prevalence of GAD65 AAb positivity in this study was 51.3 %, with a median disease duration of 7 years. This frequency is similar to two other South African studies performed in black participants i.e. 44 % [24] and 60 % [25], in both of which disease duration was less than that of the current study – mean of 3.4 years and median of 5 years, respectively. However, it is lower than the GAD65 AAb frequencies seen in newly diagnosed White (European and American; 72–76 %) [26, 27], Japanese (60–70 %) [28], and African American (56–76 %) [27, 29] populations with T1D. Similarly, the prevalence of GAD65 AAbs was higher for South African white participants (median disease duration of 8.5 years) at 66 % [25]. In addition, the GAD65 AAb prevalence seen in our study is higher than that seen in other sub-Saharan countries, namely Cameroon (34 %, disease duration: <5 years) [30] and Tanzania (30 %, median disease duration of 3 years) [31].

Similarly, in our cohort we found the prevalence of IA2 AAbs (12.8 %) to be lower than the frequencies seen in newly diagnosed white (European: 58 % and American: 63 %) [26, 27], Japanese (60–65 %) [28], and African American (42 %) [27] populations with T1D. In addition, higher IA2 AAb frequencies (41 %) were seen in a white South African population (median disease duration 8.5 years) [25]. However, the IA2 AAb frequencies noted in our study were similar to that reported in black South Africans (19 %, median disease duration of 5 years) [25], Tunisians (21 %, median disease duration of 3 years) [31] and Cameroonians (6.4 and 10 %; median disease durations < 5 years and 23 days, respectively) [30, 32].

It is clear that the frequencies of AAbs vary greatly in different populations but tend to be lower in black African populations. The exact mechanism through which this occurs is unknown, however, Padoa et al. [24] hypothesised that the autoimmune response in black participants with T1D may be attenuated compared to other populations, thus resulting in a decreased frequency of AAbs and an older age at diagnosis in the black population.

The presence of ZnT8 AAbs has been associated with a younger age at diagnosis in European, American and Chinese studies [4, 8, 12, 17]. In contrast, a British study found ZnT8 positivity to be associated with an older age at diagnosis while no association was seen in a study on newly diagnosed Czech children [14]. In our cohort, the association of ZnT8 AAb positivity with an earlier age at diagnosis was lost in multiple regression analysis with only IA2 remaining associated with an earlier age at diagnosis. The lack of association of ZnT8 AAb positivity with age at diagnosis is consistent with a study in newly diagnosed Czech children [25] and with the hypothesis that these AAbs are not disease-causing but rather reflect progression of the autoimmune response [26]. IA2 AAb positivity is associated with a more rapid progression to disease and are more commonly found in younger vs. older participants [17, 27]. However, in contrast to our study, Steck and colleagues [28] found that the presence of IA2 was not associated with age at diagnosis.

Individuals with two or three AAbs were found to have an earlier age at diagnosis than participants with either one or no AAbs. This is in agreement with a study which found that increasing number of AAbs in Italian adult participants with autoimmune diabetes were associated with a younger age at diagnosis [29]. This is likely due to an increased immune response resulting in a more rapid destruction of pancreatic β-cells and thus a decreased time to T1D onset.

Increasing duration of T1D has been shown to be associated with declining levels of ZnT8 AAbs [9, 14, 16, 30]. It is possible that the decline in AAb positivity over time is due to the continuous destruction of pancreatic β-cells. As the β-cells are destroyed, fewer antigens are presented to B cells and hence fewer AAbs are produced [31].

The limitations of this study were a relatively small sample size, lack of GAD65 and IA-2 AAb data for the entire cohort and a small proportion (12.2 %) of newly diagnosed (< 1 year) cases. In addition, this was a cross-sectional study and therefore causation could not be tested and HLA genotyping was not performed in this study. In previous investigations ZnT8 AAb positivity was not associated with the HLA DR4/DQ8 haplotype in a Finnish population [11] and in British participants from the Type 1 Diabetes Genetics Consortium (T1DGC) [14]. However, a larger study on participants from the T1DGC (Asia-Pacific, European, North American, and Britain) found an association with the HLA DRB1*04:04 allele but not with the DRB1*04:01 allele [9], an association that was not observed in the earlier study restricted to the British T1DGC participants [14]. The frequency of DR alleles varies amongst different ethnic groups [32]. Thus, future studies are necessary to determine if the low ZnT8 AAb prevalence seen in our study is due to variations in HLA DR allele frequencies in the South African black population compared to those seen in other populations.

## Conclusions

In conclusion, ZnT8 AAb prevalence is significantly lower in black South Africans with T1D than in European and African American cohorts, but similar to that in other sub-Saharan African populations, suggesting that the autoimmune destruction process may be similar in different populations but the severity of the response may be weaker in the South African black population. Futhermore, the low ZnT8 AAb prevalence in our cohort suggests that this AAb may not be useful in identifying individuals at risk for T1D in this population, but this requires conformation in longitudinal studies.

## Data Availability

The datasets used and/or analysed during the current study are available from the corresponding author on reasonable request.
